# Endoscopic closure of a duodenal fistula caused by a deformable foreign body

**DOI:** 10.1093/gastro/goac031

**Published:** 2022-07-26

**Authors:** Xiujing Yu, Yaoyi Wu, Jianshan Mao

**Affiliations:** Department of Endoscopy Center, The Second Affiliated Hospital, Zhejiang University School of Medicine, Hangzhou, Zhejiang, P. R. China; Department of Gastroenterology, The Second Affiliated Hospital, Zhejiang University School of Medicine, Hangzhou, Zhejiang, P. R. China; Department of Gastroenterology, The Second Affiliated Hospital, Zhejiang University School of Medicine, Hangzhou, Zhejiang, P. R. China

## Introduction

We describe an elderly patient who suffered severe, intermittent mid-abdominal pain due to a duodenal fistula secondary to a rarely seen deformable foreign body *Dendrobium officinale* and achieved complete relief following endoscopic closure.

## Case presentation

**Figure 1.**
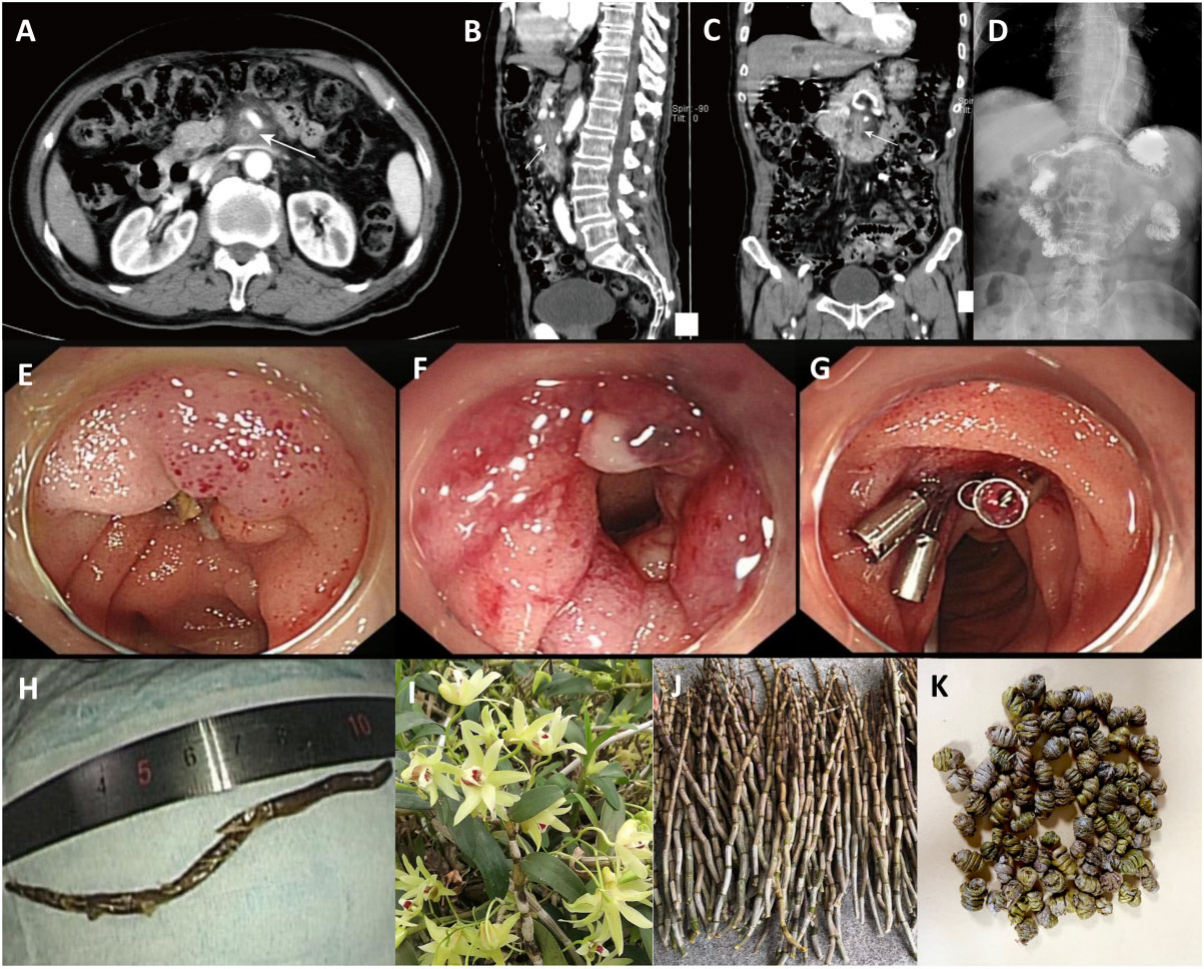
Clinical data of the case report. (A–C) The appearance of a computed tomography scan with intravenous contrast. (D) The appearance of erect radiograph from a double-contrast barium enema. (E–G) The endoscopic appearance. A stalk-shaped foreign body was lodged in a diverticulum in the horizontal part of the duodenum and surrounded by purulent liquid, forming a duodenal fistula. After an ∼10-cm stalk was extracted, it was observed that the fistula was communicating with the abdominal cavity. The perforation was fully closed with several clips. (H) A photograph of the excised specimen showed a stem of *Dendrobium officinale*. (I–K) The *Dendrobium officinale* in a natural growth state and in medicine. Stems of the fresh *Dendrobium officinale* are erect, fleshy, and knobby, but generally dried and curled into a clump for convenient storage and used as a tonic herb in traditional Chinese medicine.

A previously healthy 84-year-old man presented at the emergency department with severe, intermittent, middle-abdominal, back-referred colic pain. The patient reported that the symptoms started 1 month before arrival and progressively aggravated but did not improve with cephalosporins. The pain initially occurred during sleep and was relieved when changing positions. Neither fever, nausea, vomiting, diarrhea, abdominal distension, nor constipation was presented. He had no other medical history. His family and travel history were noncontributory. Physical examination was unremarkable except for upper abdominal tenderness and rebound tenderness. Initial laboratory work, including complete blood count, C-reactive protein, comprehensive metabolic panel, lipids, pancreatic lipase, urine routine, and stool routine, were within normal limits except for slightly decreased hemoglobin level (127 g/L; lower limit of normal, 131 g/L). The abdominal ultrasound examination identified a possible proximal aneurysm of the superior mesenteric artery with thrombosis. A computed tomography scan of the abdomen with intravenous contrast showed a tubular, progressively enhancing mass-like lesion adjacent to the superior mesenteric artery and seemly communicating with the intestinal lumen, and a thickened intestinal wall with blurred edges (Figure 1A–C). The erect double-contrast barium radiograph suggested a descending duodenal diverticulum (Figure 1D).

**Figure:**
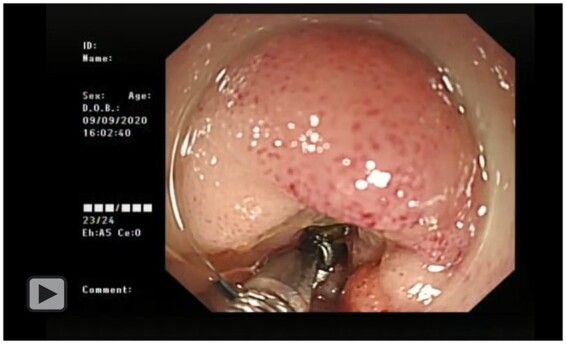
Endoscopic procedure of deformable foreign body removal.

The upper endoscopic evaluation revealed a stalk-shaped foreign body in the horizontal part of the duodenum. It was lodged in the horizontal part of the duodenum and surrounded by purulent liquid, forming a duodenal fistula (Figure 1E). After an ∼10-cm stalk was extracted, it was observed that the fistula was communicating with the abdominal cavity (Figure 1F). Subsequently, the fistula was fully closed using several clips (Figure 1G). A naso-intestinal tube was then inserted into the jejunum to provide nutrient support combined with intravenous antibiotics and a proton-pump inhibitor after the endoscopic surgery. After further questioning of the patient’s history, the patient confessed to consuming *Dendrobium officinale* before the symptom onset, which is a herbaceous plant generally dried and curled into a clump for convenient storage and used as a tonic herb in traditional Chinese medicine that is often used to make tea or medicinal food (Figure 1I–K). The dried *Dendrobium officinale* clump that the patient had swallowed happened to be stuck in the horizontal part of the duodenum; it swelled and stretched when contacting gastric contents, leaving the indigestible stem that finally caused a fistula. Five weeks after the procedure, the patient gained a good recovery without any complications.

## Discussion

Cases of perforation or fistula due to ingested foreign bodies are frequently described in the literature [1, 2]. In previous studies, 93% of the ingested foreign bodies were toothpicks and dietary foreign bodies such as fish or chicken bones [3]. There were also other odd foreign bodies like button batteries, chopsticks, ballpoint pens, and needles [4–7]. It is the first case of a duodenal fistula caused by the caulis of *Dendrobium officinale* reported in English.

In prior studies, perforation or fistula due to ingested foreign bodies often occurs in children, the elderly, patients with mental disorders, alcoholics, and people wearing dentures [8]; and ingested foreign bodies that cause unpleasant symptoms or severe complications like gastrointestinal tract perforation are usually large, sharp, or caustic [9]. Interestingly, the patient in this study ingested an initially small and spherical foreign body under normal circumstances without mental disorder or intoxication. The dried *Dendrobium officinale* as the foreign body first appearing in a lump and subsequently expanding like a stalk also reminds us to be vigilant about the safety of swallowing deformable objects that may stretch when in contact with water. It is significant to pay attention to the history of consuming deformable objects in medical history collection.

In conclusion, it is of great clinical value as the present case report may offer some help when dealing with a gastrointestinal fistula caused by foreign bodies.

## Supplementary data

Supplementary data is available at *Gastroenterology Report* online.

## Authors’ Contributions

All authors contributed to the study conception and design. Material preparation and data collection were performed by X.Y. and J.M. The first draft of the manuscript was written by X.Y. and Y.W. All authors have read and approved the final version of the manuscript.

## Funding

This work was supported by the National Natural Science Foundation of China [grant number 81372348].

## Supplementary Material

goac031_Supplementary_Data

## References

[1] Aronberg RM , Punekar SR, Adam SI et al Esophageal perforation caused by edible foreign bodies: a systematic review of the literature. Laryngoscope 2015;125:371–8. 25155167 10.1002/lary.24899

[2] Kuzmich S , Burke CJ, Harvey CJ et al Perforation of gastrointestinal tract by poorly conspicuous ingested foreign bodies: radiological diagnosis. Br J Radiol 2015;88:20150086. 25827210 10.1259/bjr.20150086 PMC4628459

[3] Goh BK , Chow PK, Quah HM et al Perforation of the gastrointestinal tract secondary to ingestion of foreign bodies. World J Surg 2006;30:372–7. 16479337 10.1007/s00268-005-0490-2

[4] Ozokutan BH , Ceylan H, Yapici S et al Perforation of Meckel’s diverticulum by a button battery: report of two cases. Ulus Travma Acil Cerrahi Derg 2012;18:358–60. 23139007 10.5505/tjtes.2012.48742

[5] Li C , Yong CC, Encarnacion DD. Duodenal perforation nine months after accidental foreign body ingestion, a case report. BMC Surg 2019;19:132. 31500608 10.1186/s12893-019-0594-5 PMC6734462

[6] Gardner AW , Radwan RW, Allison MC et al Double duodenal perforation following foreign body ingestion. BMJ Case Rep 2017;2017:bcr2017223182. 10.1136/bcr-2017-223182 PMC5728245 29222210

[7] Toyonaga T , Shinohara M, Miyatake E et al Penetration of the duodenum by an ingested needle with migration to the pancreas: report of a case. Surg Today 2001;31:68–71. 11213048 10.1007/s005950170224

[8] Birk M , Bauerfeind P, Deprez PH et al Removal of foreign bodies in the upper gastrointestinal tract in adults: European Society of Gastrointestinal Endoscopy (ESGE) Clinical Guideline. Endoscopy 2016;48:489–96. 26862844 10.1055/s-0042-100456

[9] Gambardella C , Allaria A, Siciliano G et al Recurrent esophageal stricture from previous caustic ingestion treated with 40-year self-dilation: case report and review of literature. BMC Gastroenterol 2018;18:68. 29788901 10.1186/s12876-018-0801-3 PMC5964928

